# Supplementary figures and images for: Effect of the Intake of Solid Block Dairy Products Like Cheese on Serum Uric Acid in Children: A Preliminary Mechanistic Investigation
Source: Nutrients. 2024 Nov 12;16(22):3864. doi: 10.3390/nu16223864 (PMC11597038; doi:10.3390/nu16223864)

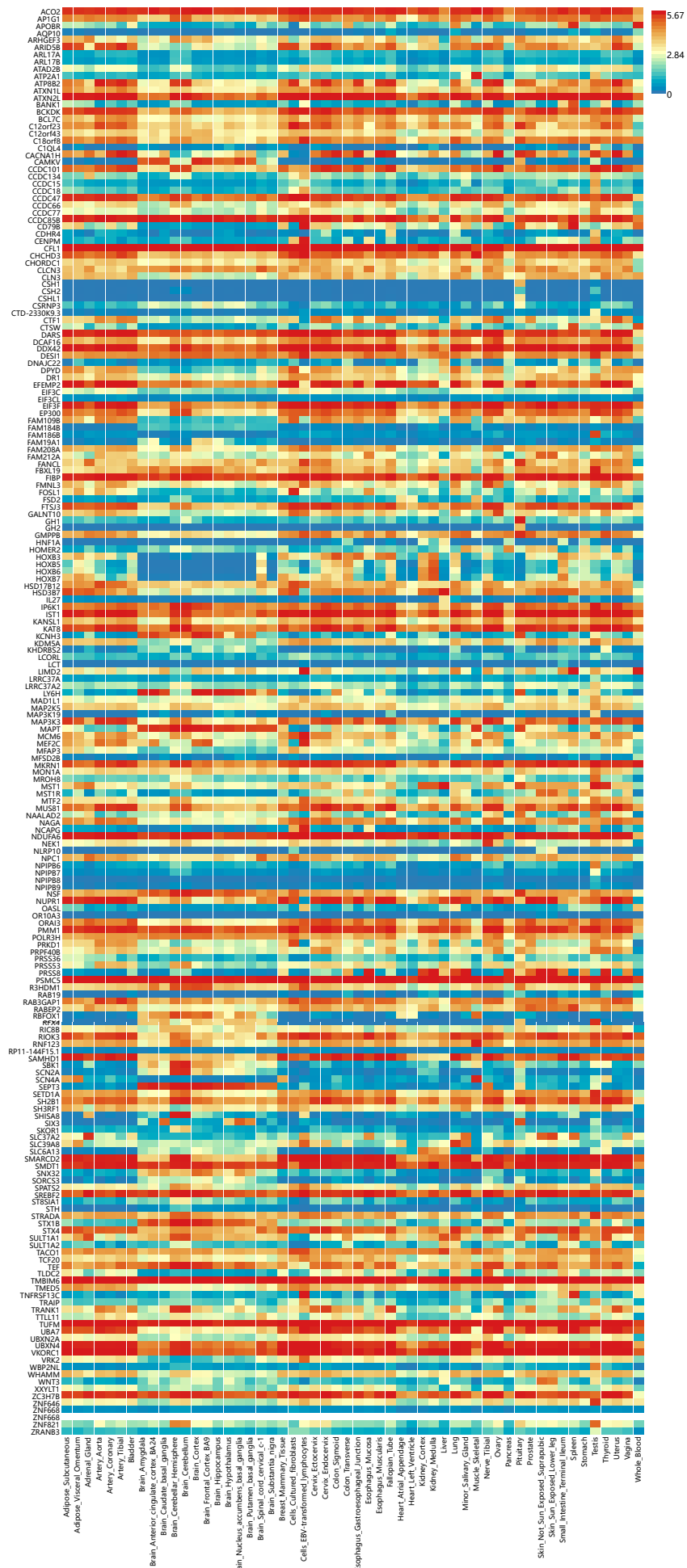

Supplement: Supplementary file 1 [file nutrients-16-03864-s001.zip › heatmap_supplement.pdf]
